# “Please let me know when I do not realize it myself”: a qualitative analysis of senior simulated patients’ experiences

**DOI:** 10.1186/s41077-019-0109-6

**Published:** 2019-07-29

**Authors:** Claudia Schelgel, Cathy M. Smith

**Affiliations:** 1 Berne College of Higher Education of Nursing, Freiburgstrasse 133, 3008 Berne, Switzerland; 2 Division of Training & Simulation, Centre for Education, Baycrest, 3560 Bathurst Street, Toronto, Canada

**Keywords:** Senior person, Standardized patient, Simulated patient, SP educator, Standards of best practice, Simulated learning environment, Safety, Patient simulation

## Abstract

**Background:**

Simulated patients (SPs), defined as being over 65 years old, are valuable partners in the training of health professionals related to the care of our aging population. Many senior SPs have been long-time members of SP programs. As SPs age, shifts in their abilities may be observed that, in turn, can affect the overall quality and effectiveness of their participation. It can be challenging and distressing for both the SP educator and the SP to acknowledge these changes and to respond in a compassionate, respectful, and ethical manner that ensures the safe and effective delivery of a simulation for all stakeholders. SP educators are looking for guidance. The aim of this study was to ask SPs from two countries (Switzerland and Canada) to identify the benefits and challenges of working as SPs as they age and to offer strategies to SP educators to accommodate and facilitate their participation.

**Method:**

A qualitative thematic analysis research design was implemented to address the study aims. A semi-structured approach with a topic guide was used to individually interview 16 SPs (9 in Switzerland; 7 in Canada). Researchers iteratively compared their results until consensus was reached in terms of identifying the themes and subthemes.

**Results:**

Three main themes, with corresponding subthemes, were identified: giving and receiving value as senior SPs, recognizing challenges when working as a senior SP, and fostering meaningful involvement for senior SPs. Meaningful involvement focused on creating a sense of security, adapting to changing abilities, acknowledging contributions, and providing opportunities to stay connected to the program.

**Conclusion:**

This study illustrates the importance of SP educators working with SPs to co-create a safe and effective work environment. Studies like this can serve as a model to provide practical strategies. Through this study, we have learned from senior SPs how we can best support them in their important work.

## Background

Globally, the number of people over the age of 60 is expected to more than double by 2050 [1]. A United Nations (UN) report [2] notes that “population aging is poised to become one of the most significant social transformations of the twenty-first century, with implications for nearly all sectors of society”. Diseases associated with aging, such as dementia, are identified by the World Health Organization (WHO) as being a major global health challenge that current and future healthcare providers must be prepared to meet [3].

Simulation is a safe way to train healthcare providers to provide effective care for older people and their families [4–9]. Simulated patients (SPs) can support the development and assessment of diverse competencies, especially those related to communication and the integration of psychomotor skills, clinical decision-making, and professionalism [10–12]. SPs are well people who are carefully trained to simulate or portray others in an authentic, flexible, and repeatable manner [13]. They may also provide feedback or assess aspects of learner performance. The scope of SP practice has evolved from representing only patients to portraying any kind of person, hence the broadening of the term SP to mean simulated participant [13, 14]. SP performance is based on theory, evidence, and practice, and the Association of Standardized Patient Educators (ASPE) has published a Standards of Best Practice (SOBP) [13].

SP programs are made up of mostly adults, including older persons, whom are defined by the WHO and UN as being, in many countries, 65 years of age or older [15, 16]. These senior SPs are valuable partners in the training of health professionals related to the care of the aging population and provide an authentic perspective [17, 18]. SP educators report that they develop deep and enduring bonds with these senior SPs, who often have been a vital part of SP programs over many years and contributed much through their involvement [19, 20].

As SP populations age, there may be increasing numbers of older SPs, just as in the larger workforce, there is a trend towards older adults remaining in the workforce rather than retiring [21]. At conference sessions presented by the researchers [22–24], SP educators have reported that as SPs age, shifts in their abilities are observed that can affect the overall quality and effectiveness of their participation. While there can be many positive effects associated with aging, there can also be less desirable changes such as a gradual deterioration of biological functions and an increase in losses associated with life transitions (e.g., retirement, death of family and friends, changing roles) [1]. Thompson et al. [17] refer to these changes in SPs as “vulnerabilities” (p7). It can be very challenging and distressing for both SP educators and older SPs to acknowledge these changes and to respond in a compassionate and respectful manner [19, 20].

SP educators are seeking guidance in how to work safely and effectively with older SPs [19, 22–24]. Although the ASPE SOBP provides principles and practices for all human role players in simulation, there are no specific considerations related to working with senior SPs. There are emerging human resource practices for working with older adults [21, 25, 26] as well as recommendations from WHO [27]. In addition, there are best practices for working with older volunteers [28]. Senior SPs have shared experiences of their practice identified in other investigations of SPs of all ages [18, 29–35]. For example, Schlegel et al. [18] undertook a qualitative study that sought perspectives from SPs on workplace satisfaction and work-related relationships with the intention that these findings might lead to a broader understanding of the SP viewpoint and provide insight and information for practical implementation. One previous qualitative study by Thompson et al.[17] specifically focused on the experiences and perspectives of older SPs and concluded that they gain a great deal from their involvement, particularly from a psychosocial perspective. Informed by the approach of Schelgel et al. [18], the aim of this study was to ask senior SPs from two countries (Switzerland and Canada) to identify the benefits and challenges of working as SPs as they age and to offer strategies to SP educators to accommodate and facilitate their participation.

## Method

### Study design

A qualitative research design [36] was adopted to allow us to explore in depth the experiences and opinions of the senior SPs related to the aims of this study.

### The setting

The Swiss SPs work at the Berne College of Higher Education of Nursing, which has over 1200 nursing students who work with SPs at all stages of their studies in both formative and assessment contexts, including Objective Structured Clinical Examinations (OSCEs). The SP Program, started in 2005, has over 100 paid SPs. Many are 65 or older because care of the aging is a strong educational focus at this institution and is reflected in the scenarios that are created. The Canadian SPs work at Baycrest in Toronto, an academic health science center fully affiliated with the University of Toronto that provides care for older adults across a variety of institutional and community-based settings [37]. The Baycrest SP program, created in 2014, is known as SAGE—Simulation Activities for Gerontological Education. Most of the 25 SPs in this program are seniors; all are volunteers. Simulations are in formative settings, and learners can include a variety of healthcare professionals, both internal and external students and staff, with a focus on team-based learning.

### Recruitment and sampling

For the study, a purposeful sampling method [38] was used to identify the subjects, who were eligible for inclusion if they were currently active SPs, at least 65 years old, and had at least a year’s experience as an SP. Altogether, 9 SPs (6 females and 3 males) in Switzerland between the ages of 65 and 83 years (mean 72.44 years) and 7 SPs in Canada (4 females and 3 males) between the ages of 68 and 88 years (mean 78.29 years) were recruited. Subjects had been SPs between 2–15 years in Switzerland and 1.5–4 years in Canada (Table 1).

**Table 1:** Demographic characteristics of SPs (total *n* = 16)

Demographic characteristics	Swiss SPs (*n* = 9)	Canadian SPs (*n* = 7)
Gender		
• Male	3	3
• Female	6	4
Age		
• Age range (years)	65–83	68–88
• Mean age (years)	72.44	78.29
Years of experience as an SP		
• Range (years)	2–15	1.5–4
• Average (years)	7.44	3.29

### Research team

The authors, CSc and CSm, collaborated to explore this topic because of their increasing involvement and shared interest in working with senior SPs. Together, they have over 45 years of experience as SP educators. CSc has a nursing background and is developing SP methodology within nursing education in Switzerland. CSm, from Canada, has a humanities and dramatic arts background and works in diverse educational and assessment contexts, including as a simulation consultant at Baycrest, where she leads the SAGE program.

### Data collection

Data was collected between November 2017 and November 2018 through in-person, semi -structured 30–60-min interviews with each individual SP, using a topic guide (Table 2). Interviews were audio recorded. Interviewers were trained to conduct this type of interviewing according to a written protocol. The Swiss interviewers were experienced, while in Canada, one interviewer was experienced and one was new to this kind of interviewing. The interviewers were not involved in working with the SPs to allow for SPs to feel comfortable voicing their perspectives. The topic guide was piloted first in Switzerland and was influenced by the questions related to the previous study conducted by CSc [18]. In Canada, a question was added about the benefits of working as an SP since this topic had been addressed by the Swiss SPs without prompting. The guide was forward and backward [10] translated from German into English for use in Canada. The topic guide was open-ended, allowing respondents enough scope to talk more deeply about their experience beyond the questions asked. Interviews were conducted in German (S = Switzerland) and English (C = Canada) and transcribed by external assistants to allow researchers to read, analyze, and interpret the information contained in the interviews [39]. Then, each researcher (CSc, CSm) checked for the accuracy of the transcripts from their respective sites. The interviews were transcribed only into the language of the interview. Follow-up interviews were conducted with some of the subjects (three in Switzerland and three in Canada) to explore certain topics that were discussed in their first interviews in greater depth, including benefits of working as an SP, strategies for memorization, the influence of the SP educator, and additional suggestions for SP educators to effectively train senior SPs.

**Table 2:** Topic guide for semi-structured interviews

Question number	Question
1	How long have you been working as SP?
2	What are some of the benefits of the work that you have done as an SP?
3	Do you recognise any challenges or changes because of your age related to:
	a. Learning the role?
	a. Performing the role?
	b. Giving feedback?
4	What would you like the SP educator to do to help you with these challenges?
5	What else would you like to tell us?

Gender and age were documented on the informed consent document

### Data analysis

A thematic analysis following the approach of Braun and Clarke [40] was adopted to identify prominent themes and subthemes. Each researcher read the transcripts of the interviews with the SPs at their respective sites, line-by-line. They independently and inductively analyzed the transcripts to search for meanings and patterns. CSc read all the transcripts (as she is fluent in both German and English) and provided an English summary of her analysis of data generated by the Swiss SPs for CSm. Over several meetings (primarily video conferencing), CSc and CSm compared their analysis, until they achieved consensus of themes and subthemes.

### Ethical approval

This study was approved by the Baycrest Research Ethics Board in Canada. An application for ethics approval was made by The Berne College of Higher Education of Nursing to the State of Berne Ethical Committee in Switzerland, but research studies reporting perceptions of employees do not require approval from the Ethical Committee of the State of Berne, Switzerland.

## Results

Analysis of the transcripts of the interviews with senior SPs identified three main themes—giving and receiving value as a senior SP, recognizing challenges as a senior SP, and fostering meaningful involvement for senior SPs—with corresponding subthemes.

### Theme 1: giving and receiving value as a senior SP

#### Contributing to training for senior care

Overwhelmingly, senior SPs report they feel great worth in helping to train health professionals who are, or will be, taking care of older adults. For some of these SPs, this work is quite personal as they have had close family members who either have died or are suffering from illnesses, like dementia, that they are being asked to portray. They recognize that they can draw on their contexts and transform a personal loss into an experience that will be meaningful for caregivers, patients, and their families through their work as SPs.

I feel I’m doing something worthwhile. (C3)

I’m a sister of a patient [with Alzheimer’s’] … I want to put myself in the role of my sister and get across to them how important it is for me to help her as best I can. (C6)

I like to help students, so they become better doctors. (S2)

I recognize the need for really good training in long term care facilities because we’re all going to be there one day. (C1)

I feel that the work is beneficial to others in the community. (C4)

#### Building relationships

In addition to altruistic motives, many of the SPs like the camaraderie of being part of a dedicated and committed group and have made deep connections. Mixing with people who have a similar appreciation and understanding of the work has an intrinsic motivating effect on them. Some of them experience the whole SP team as a team. Interacting with students and other subject matter experts is stimulating for them. As many SPs are retired, some miss the contacts with others that they had before, when they were employed, and their SP work fills a gap.

Working with some really fabulous people who are dedicated and committed is a great sense of satisfaction. (C1)

Mixing with people who are similarly enjoying in what we are doing together… (C3)

I feel we work as a team. (C6)

For me it is important to meet other people and do something meaningful. My husband has dementia and I sometimes am glad to get out of the house and see something different and meet other people. It brings me to another world for a while. (S6)

Some, however, note that although they are retired, they are very busy with many other activities in their lives and that they make the choice to prioritize doing SP work.

I really am very busy all the time. (C1)

Three times a week is too much. (C3)

The good thing is, I can choose the days of my employment. You know I have grandchildren and sometimes I have to take care of them. (S5)

SP work is described in playful terms as being fun.

I find it fun and the participants are really just having fun (C3)

We just have a good time. (C2)

Senior SPs also appreciate being part the growth of a SP program and supporting other SPs to grow and flourish while doing this work.

I have a great satisfaction in having played a small part in making this such a success. (C1)

#### Enhancing personal well-being

SPs note that the work is good for their sense of well-being, on many levels. On a very fundamental level, one SP notes simply that working as an SP “makes me happy” (C6). They see great value in this work helping them to maintain or develop cognitive functions such as memory, focus, and concentration. Regular scheduling is cited as being important to maintain these functions.

For me, being an SP is good to train my memory. (S4)

Role-training is a good help for me to concentrate. I am a person who does not focus while reading. When I prepare for a role I have to focus. This is a good exercise for me to focus. (S6)

To be employed on a regular basis helps me not to forget. (S7)

These senior SPs recognize that one day, they may face cognitive and physical challenges and through doing this work, they may be preparing learners to care for them. They also have gained positive and profound insight into and understanding about situations in their own lives.

I just feel that I’m giving back but I’m gaining, I think, even more. (C5)

I hope if I end up in a facility that somebody will care and try to understand how I present myself. (C6)

I see my mother, who I adore, and I feel, I just feel so close to her. (C5)

It’s almost self-serving, in that it’s for your mental health too. (C7)

They also note that they appreciate the acknowledgment for the work that they do

I’m told I do a good job, so it makes me feel good. (C6)

I feel I’m doing something worthwhile. I actually had a chance to see that in person. (C3)

#### Learning continuously

Some of the senior SP`s report about how multi-layered and stimulating the opportunities are for learning, related to diverse areas such as educational and theoretical processes, role playing, and health topics.

… to actually see the theoretical framework behind it put into action, I just find it incredibly interesting. (C1)

They declare that the work they do is almost self-serving. With every training and encounter, they learn and grow, be it from the SP educator or the process of learning how to be an SP. There is an intensity and focus to their commitment.

I’ve a message to portray and I want to do it properly to get that message across. (C6)

I learn a lot about illnesses, it is a win-win situation for me. (S4)

Some SPs note that they have applied their learning to other contexts such as when they do other volunteer work and they use communication skills they have observed being taught to learners.

I find that I actually use some of the things that we are teaching them, I use it myself because I am a volunteer visitor. (C3)

### Theme 2: recognizing challenges as a senior SP

#### Changing cognitive functions

The senior SPs recognize changes that are occurring that compromise their ability to work as SPs. Memorizing lines is one of the main challenges commented on. It is a constant concern and causes much worry, stress, and even fear.

I was kind of fearful of coming because you know, if you have to memorize lines, that you know, at this stage in my life I have trouble memorizing things. (C2)

I was concerned having to remember lines and stuff. (C7)

When reading and training for new roles, they note that they need to concentrate more than before and therefore need more time to prepare and more notice to participate in a session. They also do not appreciate the distraction of too much talking or a lack of clarity during training.

Assignments of short notice are a problem for me because I get stressed and I cannot concentrate. If I get the role script some time in advance, then I can prepare better for the task. (S3)

I do not want to have a lot of stress and discussions. Discussions irritate me a lot. (S2)

Concentration is also affected if they do not get regularly scheduled breaks during a simulation, even between individual encounters.

I also need breaks between encounters. Without a break it is difficult to concentrate. (S2)

#### Changing stamina levels

Senior SPs also perceive physical challenges, which they directly articulate.

We’re all older people and … I mean it’s the challenges of normal aging. Physical disabilities that start as you get older. (C1)

We are both over 80 years and we could feel how tired we get to be an SP. (S2)

Tasks of daily life become more daunting. Some of the senior SPs no longer drive and so can face long, potentially tiring journeys on public transit, traveling to do SP work.

…the only thing…well … I’ve got a long trip on the bus. (C4)

SPs also feel exhausted when they simulate a role for too long at one time or when they are asked to do too many sessions in a week. During an actual simulation, they need more recovery time between encounters.

Being asked for too many different sessions in a week is a challenge for me. (C3)

After two hours encounter with students it is enough for me, I get tired. It was not like this 15 years ago. (S2)

#### Feeling insecure

Senior SPs indicated that they feel insecure when they do not hear from the SP program for a long time, or if communication is inconsistent. They make assumptions that these gaps mean that they are no longer part of the program or that they have done something wrong.

… sometimes [there are] large gaps of time between one project and another. And I sort of wonder whether the SP program is still continuing, with me personally. (C4)

It is hard, if there are suddenly no more assignments anymore. (S4)

### Theme 3: fostering meaningful involvement for senior SPs

While analyzing the transcripts of the interviews, it became apparent that senior SPs have many suggestions for the kind of support they need from the SP educator.

#### Creating a sense of security

The senior SPs consistently point out the importance of the SP educator in creating a sense of security for them to work. Key qualities repeatedly articulated include empathy, clarity, compassion, and respect. They appreciate when SP educators recognize and then treat them as individuals, with strengths and weaknesses.

… an empathic SP trainer who understands me and trains and explains in my tempo. (S3)

… they understand that we’re learning and they’re very compassionate people, patient people. And they respect each individual, you know some of us have strengths in certain areas and some of us have strengths in other areas and they work with those strengths. They treat me as an individual and we are all learning. (C6)

Regular, constructive feedback is desired as it helps them develop their skills. If there is an issue with performance, the SP would like to be told about it in a transparent and respectful manner.

Regular feedback from the SP Trainer is an orientation for me about my performance. (S2)

If we aren’t doing it the way they want us to get the point across to the students they tell us in a very nice, constructive way. And we do it again and that’s okay with me. (C3)

The worst one SP can imagine is no longer being asked to work and not knowing why this has happened. There is a profound sense of disconnection and loss.

Please let me know when I do not realise it myself. It is a bad feeling just to be left out. (S4)

SPs would like additional sources of feedback such as video, so they can critique and learn from their performances, discuss issues with the SP educator and, hopefully, remedy the situation.

I need proof if I do not perform well anymore, like a video of my performance so we can discuss it. (S5)

I think what [looking at videos] does, is it provides the opportunities for discussion - like could I have done it differently or could I have done it better… just throw it open to the group. (C1)

#### Adapting to our changing abilities

Senior SPs offer practical suggestions for SP educators to adjust to their changing cognitive and stamina levels such as creating a clear script, developing scenarios that do not have to be memorized, creating key words that are easy to recall, providing videos of actual patients that allow them to model their performance and make it easier to remember key details, avoiding unnecessary and distracting discussions in training, creating routines, and providing breaks. They also appreciate being able to choose how long they will participate in a session.

A clear script would be good, so one can follow it easier. (S6)

Regular tasks such as being cast in the same case help. You get a routine, and this helps you not to forget. (S3)

I like to know early enough when I have my tasks. (S8)

… most of the things we do – the memory work is basically looking over notes and just remembering what your role is and who you’re talking to and so forth, but we’re not memorizing… (C3)

I really like that I can choose the length of my encounter myself. (S1)

… there’s less lines and I can use more of my own personality. (C6)

A strong structure with flexibility is welcomed. Clarity and thoroughness of instruction is important.

It’s highly structured but it’s organic at the same time… it’s an exterior structure with an interior kind of latitude. (C1)

I find that, first of all the SP educator is very thorough in her instructions. (C3)

#### Acknowledging our contribution

There is a desire to have their work acknowledged in public by their peers, the educational community, and family members.

People want to see their work more often…what they want to do is [say]: Look, I’m in five videos, here I am. Look what I’m doing … when I am here. (C1)

They also want to know about and see how their work is applied, such as one SP who had a profound moment sitting in a class and watching videos that they had been involved in making.

This was the first time that I had actually seen the good I was doing … it was actually being used and being useful. (C3)

There is also an interest in knowing how their work fits into the larger context.

Something I would be interested in … would be to look at where [our] work as an SP fits in with the whole program. (C1)

#### Providing opportunities to stay connected

Senior SPs would like to stay connected to the program, and some express a desire to do this work as long as they can. When they are no longer active SPs, they still would like to be invited by for special events.

I hope there will be more to come. (C2)

I just keep doing it as long as I can get on the bus. (C3)

I am interested in what’s going on, even when I am no more a SP. (S6)

Table 3 provides a comprehensive summary of concrete strategies from senior SPs for SP educators to consider when working with them. There are suggestions connected to each of the four subthemes related to the third theme of fostering meaningful involvement for senior SPs.

**Table 3:** Summary of strategies from senior SPs for SP educators related to theme 3: fostering meaningful involvement for senior SPs

Create a sense of security	• Be supportive. • Be empathic. • Be compassionate. • Be transparent. • Be patient. • Have a sense of humor. • Make it fun. • Treat us as individuals. • Be structured yet responsive to the needs of the group. • Adjust your training style when needed (e.g., work with us on a one-to-one basis if we are struggling). • Facilitate our autonomy within a given structure. • Provide feedback, especially if something has not gone well. • Contact us if we are not performing according to expectations. Give us a chance to fix it. • Feedback supported by video can be the most helpful proof for us to see and hear how we are doing. • Create a culture of openness so we can identify areas of strength and disclose any concerns (e.g., not being able to memorize something the way we used to). • Let us turn down work if we are not up to it or do not want to do it without fearing this choice will affect our standing in the program.
Adapt to our changing abilities	• Provide thorough instructions. • Provide clear information. • Simplify the details in the scenario. • Avoid unnecessary discussions/distractions during role training. • Develop scenarios that do not have to be memorized. • Provide “buzz” words (e.g., words that are easy for us to remember within the script). • Allow us to draw on our own experience to fill in certain role details, if possible. • Create routines that help us to remember (e.g., give us the same role). • Provide alternative ways for us to work (e.g., trigger videos) that do not stress us or tax our memory. • Give us simpler scenarios if you notice that we are struggling to memorize things. • Send out the invitation for our next assignment well ahead of time. • Recognize that we need a longer time to prepare than we used to. • Do not give us too many assignments in too short a time. • Let us choose how often and how long we work. • Two hours is a good length of time for our involvement in a session, especially if we must give feedback. • Giving feedback gets difficult when we work with too many students in a row during a session. • Do not ask us to come in too often.
Acknowledge our contributions	• Tell us when we do a good job. • Provide us with an overall summary of where our work is being used. • Let us know the impact of our work. • Invite us to sessions where our work is being. used/discussed (e.g., at grand rounds) so we can see the impact of our work. • Show us how the work we are doing fits into the larger educational context.
Provide opportunities for us to stay connected	• Find even more ways for us to contribute. • Provide opportunities for us to develop our skills. • Provide more opportunities for us to network/build relationships with each other. • Keep in regular contact with us (e.g., newsletter) even if we are no longer active. • Let us know if there are lags in work so we do not think that you have forgotten about us or the program has stopped running.

## Discussion

The aim of this study was to ask senior SPs from two countries (Switzerland and Canada) to identify the benefits and challenges of working as SPs as they age and to offer strategies to SP educators to accommodate and facilitate their participation. As previously mentioned, although senior SPs shared experiences of their practice identified in other studies of SPs, here, we focus on those relating to their seniority. Three themes were identified: giving and receiving value as a senior SP, recognizing challenges as a senior SP, and fostering meaningful involvement for senior SPs.

SPs in both Switzerland and Canada noted that they derived great value from their work as SPs. They have a strong desire to contribute to the education of healthcare providers, specifically related to the care of older persons. Part of their desire is altruistic, and part of it is pragmatic. They want to make sure that if the time ever comes, and they need to be cared for, caregivers will be prepared. They also value the opportunity to enhance their well-being, enjoy themselves, learn, further develop their skills, and create new social networks. Thompson et al. [17] noted similar findings in their earlier study, done in the UK. In this study, SPs also recognize that their involvement in SP work can help them maintain or improve their cognitive functions. These findings of positive biopsychosocial motivators for what engages seniors in work are congruent with many of those contained in the WHO World Report on Aging and Health [27], as well as in human resources [26] and volunteer practices related to working with older adults [28].

Moreover, even though the Swiss SPs are paid, compared to the volunteer SPs at Baycrest, it appears that money is not the primary incentive for them. This finding aligns with Herzberg’s two-factor theory [41] based on the assumption that there are two sets of factors that influence motivation in the workplace: hygiene factors, which are extrinsic and linked to elements such as compensation and working conditions, and motivators, which are linked to the intrinsic motivation of the job itself, including recognition, achievement, and opportunities for growth. From our study, it appears that senior SPs highly value these intrinsic motivators whether they are paid or not. Although the aim of this study was not to focus on what motivates senior SPs, during the interviews, the SPs repeatedly articulated these motivators as being significant reasons for their involvement in SP work. SP educators can benefit from understanding, recognizing, and leveraging these intrinsic self-identified motivators, when engaging with senior SPs. For example, the WHO report [27] notes that “older workers are especially able to grasp difficult situations and then concentrate on vital tasks”(p52). Some of the senior SPs in this study illustrate quite vividly these abilities when they describe how they portray patients with dementia, while drawing on experiences with family members who have suffered from this disease. At the same time, they keep in mind the bigger purpose for doing this work of contributing to the improvement of healthcare for older people. It should be noted that senior SP participation in roles that could be potentially traumatic for them is not a mandatory part of their participation. They always are at choice. There are policies in place at both sites related to informing SPs about the type of role they are being asked to portray and at any point in the process, if they feel uncomfortable, they can choose to step out of their involvement. In addition, they are de-roled and debriefed [42, 43] after a simulation session and are welcome to contact the SP educator if they need further debriefing or support. These strategies are important for maintaining the psychological safety of these senior SPs and are examples of the implementation of the ASPE SOBP, domain 1—safe work practices [13].

The key challenges these SPs identified as they age are primarily related to their perceptions of changing cognitive and physical abilities and the resulting stress felt from the impact on performance. These changes align with those noted by WHO [1], such as the fact that fading cognitive and physical abilities can lead to social isolation. It is important, however, that SP educators take care not to make assumptions about the abilities of senior SPs solely based on their age. As WHO [1] notes, “these changes are neither linear nor consistent, and they are only loosely associated with a person’s age in years.” In this study, senior SPs had a high degree of insight about these changes and a willingness to disclose them. The perception that a lack of consistent communication on the part of a program could cause some SPs to feel insecure and/or feel that they are no longer wanted or needed is a finding that the researchers had not anticipated. This perception is a reminder that, as the Thompson et al. [17] study notes, for many of these senior SPs, the “engagement seems to have had a deep significance for them” (p14) that cannot be underestimated or taken for granted. The findings of the Baycrest Research About Volunteering in Older Adults (BRAVO) study, also emphasize the powerful biopsychosocial health benefits associated with senior participation in volunteer work [28]. Indeed, the Thompson et al. [17] study recommends a holistic solution that SP programs with older people be developed not only “as a learning strategy for students, but also as a means of promoting the well-being of the older SPs” (p16).

Senior SPs indicate they can thrive and contribute when SP educators bear in mind changes and challenges. They offer numerous strategies to foster their meaningful involvement related to creating security through effective communication, addressing their changing abilities, acknowledging their involvement, and keeping them feeling connected. Key to the quality of their involvement is working with an SP educator who is empathic, supportive, transparent, and honest with them. These findings highlight the importance of the role and responsibilities of the SP educator and align with many of the principles and practices outlined in the ASPE SOBP, particularly related to domain 1, safe work environment, and domain 3, training [13]. Principal 1.1, safe work practices, describes the importance of ensuring safe working conditions in the design of a SP activity (e.g., number of rotations, number of breaks, physical, cognitive, and psychological challenges in the role portrayal). The physical and psychological safety of aging SPs are imperative. Paul O’Neill [44], a business leader who has championed both worker and healthcare safety, developed three questions related to workers feeling safe in a workspace: “Am I treated with dignity and respect by everyone I encounter?” “Am I given the knowledge, tools and support that I need in order to make a contribution to my organization?” “Did somebody notice I did it?” As is demonstrated in this study, only when senior SPs can answer “Yes” to these three questions, can the SP educator assume that senior SPs feel secure, which is a desired condition for a high-quality performance as well as personal wellness.

The practical and proactive strategies the older SPs provide for SP educators that are contained in Table 3 could be drawn on as adjunct contextual examples of applications of the principles and practices of the SOBP with the proviso that there is no one right way to apply the SOBP. These suggestions point out that SP educators must adapt the SOBP to the SPs rather than the other way around. Borrowing from the philosophy of the patient engagement movement [45] which focuses on “shifting the clinical paradigm from determining ‘What is the matter?’ to discovering ‘What matters to you?’” (p. 1627), this study suggests that senior SPs can and should be engaged as collaborators in designing and managing their work as SPs.

## Limitations

There are some potential limitations to this study. The sample size is small, and the study was conducted only in two countries with many cultural similarities. The interviews were conducted in two languages, and the transcriptions were done in the language of each site (German and English). Budget restrictions prevented the translation of the German interviews into English. Only CSc could read both languages. However, this is part of the challenge of doing international research. The researchers took account that their close relationships with the subject SPs might affect the analysis and so strove to keep going back to the data, to reflect on their responses, and to check in with each other through repeated meetings. Although the subjects were de-identified, and a neutral person interviewed them, there is always the possibility SP responses were colored by a concern that they say the “right” thing in order to remain part of the program or not upset their relationship with the SP educator or other SPs in the program. There is also the consideration that the work demands of the programs are very different. The Baycrest SPs currently work in only formative educational settings, often create improvised videos, and usually do not provide feedback while the Swiss SPs participate in both face-to-face educational and summative assessment sessions and often give feedback. Given these fundamental differences in work demands, their responses still had much in common. Finally, senior SPs were not part of the research team for this study, and as noted by other researchers [35], SPs are key stakeholders in this type of study and should be considered for inclusion as part of the research team for future studies related to aging SPs.

## Conclusion

Senior SPs provide an authentic perspective and presence in healthcare education and assessment. SP educators have an ethical responsibility to understand and adapt to specific challenges related to working with senior SPs and ensure their on-going well-being [43, 46]. Future research might also examine the most effective, safe, and ethical ways to engage with senior SPs in both formative and summative contexts. Awareness of implicit bias related to ageism and learning how to ascertain if a person has decreasing cognitive or physical abilities are important considerations for an SP educator when working with senior SPs. It may be valuable to extend this work to gather the perspectives of senior SPs from many different countries and cultural perspectives to see if these findings resonate in other countries and contexts and if any other themes and suggestions can be identified. This process could also serve as a model for looking at other specific populations of SPs who may require special consideration, such as children, adolescents, volunteers, and vulnerable or underserved populations. Through this study, we have learned from senior SPs how we can best support them in their important work.

## Data Availability

The datasets used and/or analyzed during the current study are available from the corresponding author on reasonable request.

## Abbreviations

ASPE: Association of Standardized Patient Educators
BRAVO: Baycrest Research About Volunteering Among Older Adults
OSCE: Objective Structured Clinical Examination
SOBP: Standards of Best Practice
SP: Simulated/standardized patient/participant
UN: United Nations
WHO: World Health Organization
